# Levels of Angiopoietin 2 Are Predictive for Mortality in Patients Infected With Yellow Fever Virus

**DOI:** 10.1093/infdis/jiad389

**Published:** 2023-09-15

**Authors:** Cornelia A M van de Weg, Mateus V Thomazella, Mariana P Marmorato, Carolina A Correia, Juliana Z C Dias, Alvino Maestri, Luiz G F A B E Zanella, Natalia B Cerqueira, Alvina C Félix, Carlos H V Moreira, Renata Buccheri, Priscilla R Costa, Esper G Kallás

**Affiliations:** Medical Investigation Laboratory 60, School of Medicine, University of São Paulo, Brazil; Department of Internal Medicine, Erasmus Medical Center, Rotterdam, the Netherlands; Medical Investigation Laboratory 60, School of Medicine, University of São Paulo, Brazil; Medical Investigation Laboratory 60, School of Medicine, University of São Paulo, Brazil; Medical Investigation Laboratory 60, School of Medicine, University of São Paulo, Brazil; Medical Investigation Laboratory 60, School of Medicine, University of São Paulo, Brazil; Medical Investigation Laboratory 60, School of Medicine, University of São Paulo, Brazil; Medical Investigation Laboratory 60, School of Medicine, University of São Paulo, Brazil; School of Medicine, Hospital das Clínicas, Sã‌o Paulo, Brazil; Institute of Tropical Medicine, University of São Paulo, São Paulo, Brazil‌; Infectious Diseases Institute “Emílio Ribas”, São Paulo, Brazil‌; Department of Infectious and Parasitic Diseases, School of Medicine, University of São Paulo, Brazil; Department of HIV, Infectious Diseases and Global Medicine, University of California, San Francisco; Zuckerberg San Francisco General Hospital, California; Infectious Diseases Institute “Emílio Ribas”, São Paulo, Brazil‌; Medical Investigation Laboratory 60, School of Medicine, University of São Paulo, Brazil; Medical Investigation Laboratory 60, School of Medicine, University of São Paulo, Brazil; Department of Infectious and Parasitic Diseases, School of Medicine, University of São Paulo, Brazil

**Keywords:** angiopoietin 2, endothelial damage, yellow fever

## Abstract

In 2018 there was a large yellow fever outbreak in São Paulo, Brazil, with a high fatality rate. Yellow fever virus can cause, among other symptoms, hemorrhage and disseminated intravascular coagulation, indicating a role for endothelial cells in disease pathogenesis. Here, we conducted a case-control study and measured markers related to endothelial damage in plasma and its association with mortality. We found that angiopoietin 2 is strongly associated with a fatal outcome and could serve as a predictive marker for mortality. This could be used to monitor severe cases and provide care to improve disease outcome.

## Teaser

Markers of endothelial cell activation were measured in patients with yellow fever virus infection at the time of presentation. Extremely increased levels of angiopoietin 2 were detected in nonsurvivors vs survivors, suggesting that angiopoietin 2 could serve as a predictive marker for mortality.

Yellow fever virus (YFV), an arbovirus belonging to the Flavivirus family, has caused large outbreaks of viral hemorrhagic fever in South America in the past few years. Among those is the one of 2018 in São Paulo state, Brazil. Of all people with YFV who are symptomatic, an estimated 15% to 25% will develop severe disease with hepatic and renal failure and a hemorrhagic diathesis (reviewed by Monath [1]). The mortality rate of patients with severe YFV infection in the 2018 São Paulo outbreak was around 35% [2], and many of those patients experienced severe treatment-resistant hypotension in the days before death [1]. This and the occurrence of disseminated intravascular coagulation (DIC) suggest that endothelial cells play an important role in the pathogenesis of YFV infection [3].

In this case-control study, we found evidence that endothelial cells are highly activated during YFV infection and that this is associated with mortality.

## METHODS

### Clinical Cohort

Patient samples were selected from the cohort established during the 2018 YFV outbreak in São Paulo state, as described previously [2]. Ethical approval was obtained from the ethical review boards at the Infectious Diseases Institute “Emílio Ribas” and Hospital das Clínicas, University of São Paulo (CAPPesq 15477, CAAE 59542216.3.1001.0068).

In short, patients aged >18 years and admitted to 2 reference tertiary hospitals in São Paulo city with signs of YFV infection and a travel history to an area with known YFV transmission were included after written informed consent from themselves or their legal guardians. YFV infection was confirmed by detection of RNA with real-time polymerase chain reaction in the serum sample at admission or in tissue at autopsy. Plasma samples were stored at −80 °C.

For this study, we selected all available samples from nonsurvivors and matched the samples with survivors based on age, gender, and days of symptoms. For a control group, we used the convalescent plasma samples from the survivors at day 30.

### Laboratory Assays

The viral load was determined by extracting RNA on the automated platform NucliSENS easyMag (Biomérieux) and by using primers and probes, which could distinguish wild type YFV from the vaccine strain. The quantification of viral load is described elsewhere [2].

Commercially available enzyme-linked immunosorbent assays (Quantikine; R&D Systems) were used to determine the levels of angiopoietin 2 (Ang-2), soluble vascular endothelial growth factor receptor 2 (sVEGFR-2), matrix metalloproteinase 2 (MMP-2), matrix metalloproteinase 9 (MMP-9), and soluble endoglin (sENG). Every sample was run in duplicate, and the assays were performed according to the manufacturer's instructions. Repetitive freeze-thaw cycles were avoided.

### Statistics

The Kruskal-Wallis test was used to determine whether there was a significant difference in continuous variables among all the groups. If so, the Mann-Whitney *U* test with Bonferroni correction was used to determine whether there was a significant difference between the groups. A Fisher exact test was used to compare categorical variables.

## RESULTS

From the cohort of patients infected with YFV, for which data were collected between 11 January 2018 and 10 May 2018, we included all 27 deceased patients for this analysis. Next, we selected 27 survivors matched by age, gender, and days of symptoms. Because the deceased patients were all male, no female patients were included in this study.

There were no significant differences in age and days of symptoms between survivors and nonsurvivors (Table 1). There was also no significant difference in viral load between survivors and nonsurvivors at the day of presentation, although there was a trend toward a higher viral load in nonsurvivors.

**Table 1.** Demographical and Clinical Data of Patients Infected With Yellow Fever Virus

	Survivors (n = 27)	Missing Values	Nonsurvivors (n = 27)	Missing Values	*P* Value
Gender: male, %	100	0	100	0	>.99
Age, y	44 (35–57)	0	50 (36–63)	0	.37
Days of symptoms	7 (6–9)	0	7 (5–9)	0	.83
Viral load, log_10_ copies/mL	4.4 (3.8–6.3)	5	5.6 (5.0–6.4)	1	.08
Alanine transaminase, U/L	2339 (1248–2778)	4	2370 (1634–5194)	4	.77
Aspartate transaminase, U/L	2238 (856–3868)	3	6103 (3648–14.913)	4	.001
Prothrombin time, s	14.1 (11.0–16.0)	2	23.1 (17.1–36.8)	4	<.001
Creatinine, mg/dL	0.93 (0.77–1.10)	5	4.09 (2.98–5.35)	5	<.001
Count/μL					
Leukocyte	3100 (2150–4160)	2	5885 (4280–11 525)	1	<.001
Neutrophil	1470 (1185–1915)	2	4830 (3133–9078)	1	<.001
Lymphocyte	700 (530–1365)	2	815 (535–1628)	1	.019
Platelet	90 (57–109)	7	80 (46–101)	5	.50

Data are presented as median (IQR) unless noted otherwise.

**Figure 1.**
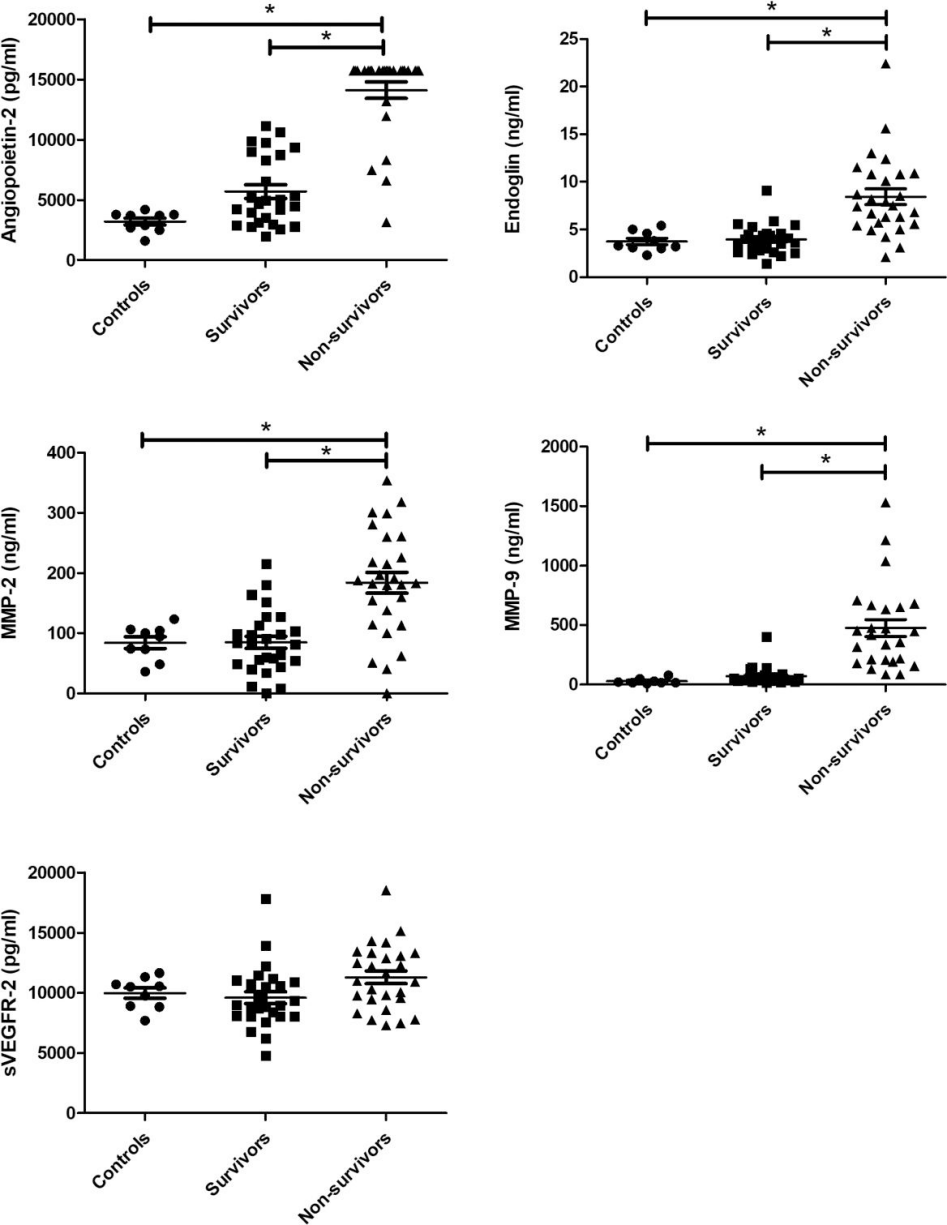
Endothelial-related markers in patients infected with yellow fever virus. Levels of angiopoietin 2, endoglin, MMP-2, MMP-9, and sVEGFR-2 were measured in the plasma samples of patients in the acute phase of infection who survived (n = 27) or died (n = 27) during the follow-up period. Patients at the convalescent phase (n = 9) were used as a control group. *P < .001, except for MMP-2 nonsurvivors vs controls (*P* = .017). Median levels were as follows. Angiopoietin 2: nonsurvivors (>15.738 pg/mL), survivors (4.816 pg/mL), controls (3.691 pg/mL). Endoglin: nonsurvivors (7.5 ng/mL), survivors (3,9 ng/mL), controls (3,3 ng/mL). MMP-2: nonsurvivors (183 ng/mL), survivors (81 ng/mL), controls (94 ng/mL). MMP-9: nonsurvivors (416 ng/mL), survivors (41 ng/mL), controls (17 ng/mL). sVEGFR-2: nonsurvivors (10.974 pg/mL), survivors (8.955 pg/mL), controls (10.508 pg/mL). MMP-2, matrix metalloproteinase 2; MMP-9, matrix metalloproteinase 9; sVEGFR-2, soluble vascular endothelial growth factor receptor 2.

Levels of Ang-2, sENG, MMP-2, and MMP-9 were all significantly elevated in nonsurvivors vs survivors and the control samples (Figure 1). There was no significant difference between samples from survivors and convalescent controls. sVEGFR-2 showed no significant difference between groups.

The median levels of Ang-2 at presentation were >3-fold higher in nonsurvivors (>15.738 pg/mL) vs survivors (4.816 pg/mL). Moreover, there was hardly any overlap in values, with only 4 samples of nonsurvivors in the same range as the survivors (Figure 1). The value of the majority of the samples (21/27, 78%) was higher than the upper limit of detection.

## DISCUSSION

In this analysis, we compared the levels of markers of endothelial cell activation between survivors and nonsurvivors at the day of admission, showing that levels of Ang-2 had the strongest association with disease outcome.

We previously determined these markers in patients with dengue virus (DENV) infection with and without signs of plasma leakage [4]. Here we also found increased levels of Ang-2 and MMP-2 in cases of DENV infection with plasma leakage vs those without. Similarly, Ang-2 proved to be the strongest surrogate marker for a compromised endothelial barrier leading to vascular leakage in our cohort with DENV.

Pathologic studies from patients infected with YFV have shown that viral antigen is mainly found in the hepatocytes of the liver [5]. In a recent study on liver samples from patients with disease, increased expression of endothelial cell adhesion molecules suggested that the endothelium is highly activated during severe YFV infection [6]. Moreover, the occurrence of hypotension and DIC indicates that endothelial cells in the whole body are affected. Finally, the damage upon the endothelial cells can be due to the systemic release of large amounts of cytokines [1] and a high plasma yellow fever viral load, as the latter was also associated with mortality, as described previously [7].

MMP-2 and MMP-9 are matrix metalloproteinases, and with their proteolytic activity they can degrade components of the extracellular matrix and increase vascular permeability. One study also showed significantly increased levels of MMP-2 and MMP-9 in patients with DENV infection and plasma leakage [8]. Moreover, MMP-2 was more strongly correlated with the occurrence of plasma leakage than MMP-9 [8]. Elevated levels of MMP-2 and MMP-9 have been detected in patients with sepsis, but they are not able to predict mortality [9].

sENG is a member of the TGF-β signaling pathway and can promote vascular leakage by inhibiting it. In our previous study, we found no difference in levels between patients with DENV infection and healthy controls [4]. However, these patients experienced only mild disease, and in a recent study, levels in patients with severe sepsis and septic shock were significantly elevated vs healthy controls [10], suggesting that increased levels of this marker are indicative for severe disease. This finding is supported by the study of Mariappan et al [11], which showed significantly elevated ENG mRNA levels in patients with severe dengue around the time of defervescence, when severe plasma leakage usually occurs.

Ang-2 is a competitive inhibitor of the Tie-2 receptor, which is abundant in vascular endothelial cells (reviewed by Akwii et al [12]). Ang-1 also signals through this receptor and induces endothelial quiescence. Binding of Ang-2 to the Tie-2 receptor can disrupt the endothelial barrier, resulting in extensive endothelial cell activation. Ang-2 is a strong predictor of mortality in patients in the intensive care unit who are critically ill [13]. Significant increased levels have also been detected in patients with dengue hemorrhagic fever during the critical stage of their disease [14].

Interestingly, in a recent study, proteomics was performed on patients who were septic with and without DIC [15]. In this study, levels of Ang-2 were significantly increased in patients with DIC vs those without. Moreover, an unsupervised proteomics analysis showed that Ang-2 was a central hub linking the processes of vascular function, endothelial inflammation, and coagulation [15]. In our cohort, there were signs of DIC in nonsurvivors vs survivors, as expressed in a prolonged prothrombin time (Table 1). The same study showed in 2 cohorts that Ang-2 was a better predictor for survival than the individual parameters of DIC. This is in agreement with our study, where Ang-2 was the strongest predictor of mortality. Moreover, the samples from the nonsurvivors were drawn, on average, 13 days before they died (IQR, 9–18 days).

This means that Ang-2 could be used as an early predictive marker for mortality during YFV infection, in which patients with highly increased levels should be assigned to intensive monitoring. Altogether, we are the first to provide evidence that endothelial cell activation highly contributes to pathogenesis during YFV infection and that Ang-2 could be used as a predictive marker for mortality.

## Notes

***Acknowledgments*.** We are grateful to all the volunteers who accepted to be part of this study and to the HCFMUSP complex (Hospital das Clínicas da Faculdade de Medicina da Universidade de São Paulo) for providing the structure to perform all assays.

***Author contributions*.** C. A. M. V. W. worked on the study design, data generation and analysis, manuscript writing and review. M. V. T., M. P. M., and C. A. C. worked on data generation, manuscript writing and review. All authors contributed to the sample collection, processing, and yellow fever cohort preparation.

***Financial support.*** This work was supported by FAPESP (São Paulo Research Foundation) to M. V. T. (grants 2016/01735-2 and 2019/13713-1) and L. G. F. A. B. E. Z (grant 2016/01735-2). and by CAPES (Coordenação de Aperfeiçoamento de Pessoal de Nível Superior) to M. P. M. and C. A. C. (PhD scholarships) and to C. A. M. V. W. (personal travel grant). Funding to pay the Open Access publication charges for this article was provided by Erasmus MC.

## References

[1] Monath TP . Yellow fever: an update. Lancet Infect Dis 2001; 1:11–20. 11871403 10.1016/S1473-3099(01)00016-0

[2] Kallas EG , D’Elia Zanella L, Moreira CHV, et al Predictors of mortality in patients with yellow fever: an observational cohort study. Lancet Infect Dis 2019; 19:750–8. 31104909 10.1016/S1473-3099(19)30125-2

[3] Bailey AL , Kang LI, de Assis Barros D’Elia Zanella LGF, et al Consumptive coagulopathy of severe yellow fever occurs independently of hepatocellular tropism and massive hepatic injury. Proc Natl Acad Sci U S A 2020; 117:32648–56. 33268494 10.1073/pnas.2014096117 PMC7768776

[4] van de Weg CA , Pannuti CS, van den Ham HJ, et al Serum angiopoietin-2 and soluble VEGF receptor 2 are surrogate markers for plasma leakage in patients with acute dengue virus infection. J Clin Virol 2014; 60:328–35. 24928471 10.1016/j.jcv.2014.05.001

[5] Quaresma JA , Barros VL, Fernandes ER, et al Reconsideration of histopathology and ultrastructural aspects of the human liver in yellow fever. Acta Trop 2005; 94:116–27. 15829426 10.1016/j.actatropica.2005.03.003

[6] Olimpio FA , Falcao LFM, Carvalho MLG, et al Endothelium activation during severe yellow fever triggers an intense cytokine-mediated inflammatory response in the liver parenchyma. Pathogens 2022; 11:101. 35056050 10.3390/pathogens11010101 PMC8779659

[7] Avelino-Silva VI , Thomazella MV, Marmorato MP, et al Viral kinetics in sylvatic yellow fever cases. J Infect Dis 2023; 227:1097–103. 36316804 10.1093/infdis/jiac435

[8] Her Z , Kam YW, Gan VC, et al Severity of plasma leakage is associated with high levels of interferon gamma-inducible protein 10, hepatocyte growth factor, matrix metalloproteinase 2 (MMP-2), and MMP-9 during dengue virus infection. J Infect Dis 2017; 215:42–51. 28077582 10.1093/infdis/jiw494

[9] Serrano-Gomez S , Burgos-Angulo G, Nino-Vargas DC, et al Predictive value of matrix metalloproteinases and their inhibitors for mortality in septic patients: a cohort study. J Intensive Care Med 2020; 35:95–103. 28931365 10.1177/0885066617732284

[10] Faiotto VB , Franci D, Enz Hubert RM, et al Circulating levels of the angiogenesis mediators endoglin, HB-EGF, BMP-9 and FGF-2 in patients with severe sepsis and septic shock. J Crit Care 2017; 42:162–7. 28746898 10.1016/j.jcrc.2017.07.034

[11] Mariappan V , Adikari S, Shanmugam L, Easow JM, Balakrishna Pillai A. Expression dynamics of vascular endothelial markers: endoglin and syndecan-1 in predicting dengue disease outcome. Transl Res 2021; 232:121–41. 33567345 10.1016/j.trsl.2021.02.001

[12] Akwii RG , Sajib MS, Zahra FT, Mikelis CM. Role of angiopoietin-2 in vascular physiology and pathophysiology. Cells 2019; 8:471. 31108880 10.3390/cells8050471 PMC6562915

[13] Kumpers P , Lukasz A, David S, et al Excess circulating angiopoietin-2 is a strong predictor of mortality in critically ill medical patients. Crit Care 2008; 12:R147. 19025590 10.1186/cc7130 PMC2646310

[14] Mapalagamage M , Handunnetti SM, Wickremasinghe AR, et al High levels of serum angiopoietin 2 and angiopoietin 2/1 ratio at the critical stage of dengue hemorrhagic fever in patients and association with clinical and biochemical parameters. J Clin Microbiol 2020; 58:e00436-19. 31941693 10.1128/JCM.00436-19 PMC7098750

[15] Higgins SJ , De Ceunynck K, Kellum JA, et al Tie2 protects the vasculature against thrombus formation in systemic inflammation. J Clin Invest 2018; 128:1471–84. 29360642 10.1172/JCI97488 PMC5873892

